# PLK1 maintains DNA methylation and cell viability by regulating phosphorylation-dependent UHRF1 protein stability

**DOI:** 10.1038/s41420-023-01667-9

**Published:** 2023-10-03

**Authors:** Yuchong Peng, Youhong Liu, Rirong Zheng, Yubing Ye, Yongming Fu, Linglong Yin, Yingxue Gao, Yuxin Fu, Xuli Qi, Tanggang Deng, Songwei Zhang, Xiong Li

**Affiliations:** 1 Key Laboratory of Clinical Precision Pharmacy of Guangdong Higher Education Institutes, The First Affiliated Hospital, Guangdong Pharmaceutical University, Guangzhou, Guangdong 510699 China; 2 Key Specialty of Clinical Pharmacy, The First Affiliated Hospital, Guangdong Pharmaceutical University, Guangzhou, Guangdong 510699 China; 3 NMPA Key Laboratory for Technology Research and Evaluation of Pharmacovigilance, Guangdong Pharmaceutical University, Guangzhou, Guangdong 510006 China; 4 Department of Oncology, Center for Molecular Medicine, Xiangya Hospital, Central South University, Changsha, Hunan 410008 China; 5 Hunan Key Laboratory of Molecular Radiation Oncology, Xiangya Hospital, Central South University, Changsha, Hunan 410008 China; 6 School of Pharmacy, Guangdong Pharmaceutical University, Guangzhou, Guangdong 510006 China; 7 School of Basic Medical Sciences, Guangdong Pharmaceutical University, Guangzhou, Guangdong 510006 China

**Keywords:** Ubiquitylation, Phosphorylation

## Abstract

PLK1 is a key serine/threonine kinase as well as a master mitotic regulator, but it has never been reported that PLK1 regulates DNA methylation. In the present study, we for the first time found that PLK1 inhibition disrupted global DNA methylation and elevated the expression level of tumor suppressor genes. Mechanistically, we found that PLK1 interacts UHRF1 protein to induce its phosphorylation at serine 265. Phosphorylation is required for the maintenance of UHRF1 protein stability by recruiting a deubiquitinase USP7. Conversely, PLK1 inhibition decreases UHRF1 protein interaction with USP7 and activates the ubiquitin-proteasome pathway, thereby accelerating UHRF1 protein degradation. UHRF1 degradation decreases the recruitment of DNMT1 to chromatin, and decreases the level of genome-wide DNA methylation, thereby elevating the expression of tumor suppressor genes and decreasing cell viability. We here presented the first report on the novel role of PLK1 in DNA methylation maintenance through UHRF1-DNMT1 pathway, and revealed a novel anticancer mechanism of PLK1 inhibitors.

## Introduction

Post-translational modifications (PTMs) are critical for normal cellular homeostasis. PTMs play a fundamental role in the regulation of signaling pathways and protein functions by maintaining protein turnover and interaction with partners [1–4]. They greatly expand the diversity and functionality of the proteome, and play a central role in numerous physiological processes [5]. Phosphorylation is one of the most common PTMs. Depending on protein kinases, a phosphate group (PO_4_) is added to the polar group R of serine, threonine and tyrosine in the substrate. Consequently, phosphorylation modifies the protein conformation when interacting with other molecules, to assemble and detach protein complexes [6, 7]. Protein phosphorylation is extremely important to regulate cellular growth, signal transduction and homeostasis. Aberrant protein phosphorylation results in diseases, including cancer [8].

PLK1 is a key serine/threonine kinase, which contains a kinase domain (KD) at the N terminus and a conserved polo-box domain (PBD) with two polo boxes at the C terminus [9, 10]. PLK1 is a master mitotic regulator, and controls almost every stage of the G2/M phase, including mitotic entry, chromatid segregation and cytokinesis. By phosphorylating CDC25, PLK1 activates the Cyclin B/CDC2 complex, which triggers mitotic entry [11]. In prophase, PLK1 shuttles to the kinetochores, thereby promoting proper chromosome alignment in mitosis [12]. At the onset of anaphase, PLK1 is localized at the spindle mid-zone, and later moves to the cytokinetic bridge to coordinate cytokinesis and cell abscission [13]. In addition to its key roles in mitosis, PLK1 is an important regulator for DNA replication. PLK1 together with pre-replication proteins are simultaneously recruited to chromatin in the S phase, and PLK1-immunodepleted extracts showed deficient DNA replication in the xenopus in vitro system [14]. PLK1 directly phosphorylates Orc2 to promotes DNA replication, while PLK1 depletion impairs DNA replication, thereby inhibiting in vitro S-phase progression in cancer cells [15]. Previous studies demonstrated that DNA replication was accompanied with genome-wide DNA methylation [16], but it remains elusive whether PLK1 regulates DNA methylation.

DNA methylation is a key epigenetic modification with a vital role in chromatin template-based processes, including transcription regulation, heterochromatin formation, genome stability, and X chromosome inactivation [17–19]. DNA methylation occurs at CpG dinucleotides and is regulated by the methyltransferases DNMT1, DNMT3A, DNMT3B [20–22]. DNMT1 is recruited by the ubiquitin-like PHD and RING finger domain containing 1 (UHRF1) through the SRA domain to the hemimethylated nascent DNA strands [23], and maintains the fidelity of DNA methylation modification [24–26]. UHRF1 as a typical oncogene aberrantly overexpresses in a number of cancer types, and contributes to cancer initiation and progression. UHRF1 overexpression suppresses the transcription of a panel of tumor suppressor genes (TSGs) by recruiting DNMT1 for the maintenance of DNA methylation [27]. In addition to gene amplification, PTMs of UHRF1 are the prerequisite for protein stability. Cyclin-dependent kinase CDK1 induces UHRF1 phosphorylation, thereby disassociating with deubiquitinase USP7 to accelerate UHRF1 protein degradation in subsequent mitosis [1]. PIM1 induces UHRF1 phosphorylation at Ser311, and promotes UHRF1 protein degradation, thereby inducing DNA hypomethylation and cellular senescence [28]. SET8 regulates UHRF1 protein stability in G2/M cell phase through methylation-mediated, ubiquitination-dependent degradation, thereby promoting DNA methylation homeostasis [2]. These data suggest that phosphorylation-dependent UHRF1 protein stability is critical for DNA methylation maintenance.

In this present study, we for the first time identified PLK1 as a key regulator of DNA methylation, and PLK1 inhibition significantly reduced genome-wide DNA methylation and elevated the expression level of TSGs. Furthermore, we clarified that PLK1 induced UHRF1 phosphorylation at Ser265, and then promoted its interaction with deubiquitinase USP7 to prevent protein degradation through the ubiquitin-proteasome pathway. Conversely, PLK1 inhibition significantly reduced genome-wide DNA methylation and decreased cell viability. These findings revealed a new molecular mechanism by which PLK1 promoted tumor progression by suppressing the expression of TSGs through UHRF1/DNMT1-maintained DNA methylation.

## Results

### PLK1 inhibition decreased the levels of global DNA methylation

**Fig. 1:**
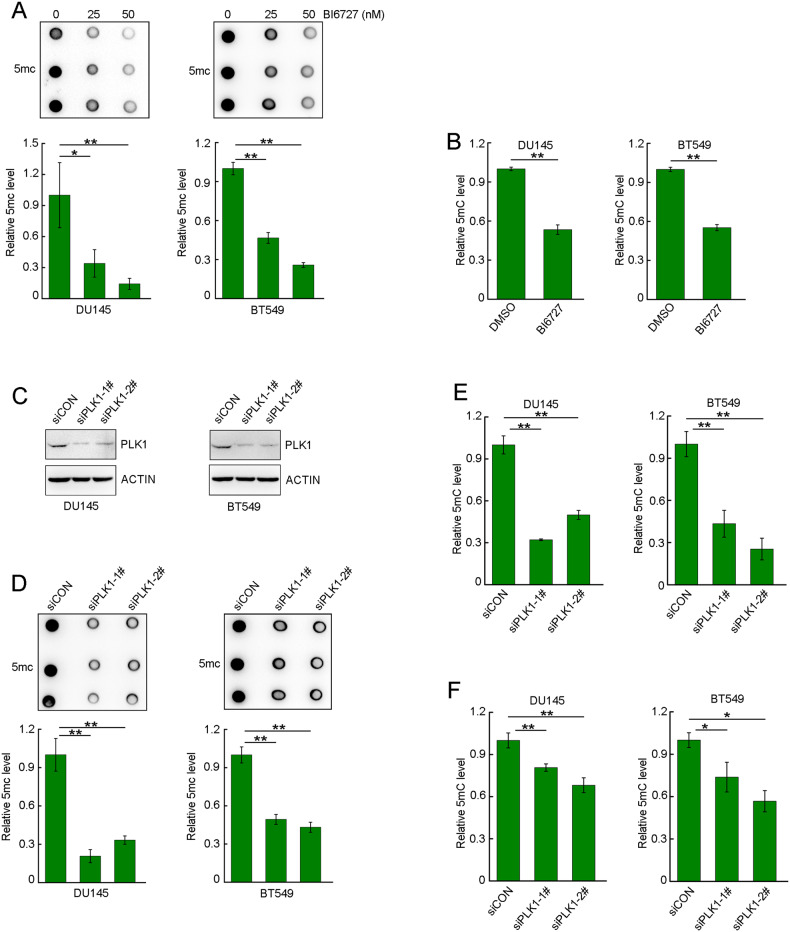
PLK1 inhibition decreased the level of DNA methylation. **A** Cells were treated with different doses of DMSO or BI6727 for 48 h, and the genomic DNA was then prepared. The levels of 5mC were assessed by DNA dot blotting, and the dot intensities were quantified. **B** Cells were treated with DMSO or BI6727 (50 nM) for 24 h, and then the genomic DNA was prepared. The levels of 5mC were assessed by ELISA assay. **C** Cells were transfected with either scrambled or two different PLK1 siRNAs for 72 h. PLK1 protein levels were assessed by western blotting. The genomic DNA of DU145 and BT549 cells were prepared, and the levels of 5mC were assessed by DNA dot blotting **D**; ELISA **E** and HPLC **F**. Data represent the mean ± SD of each group from three separate experiments. *P < 0.05, **P < 0.01.

PLK1 aberrantly overexpresses in a panel of cancer types such as breast cancer and prostate cancer, and was regarded as an ideal anti-cancer target [29]. To identify the roles of PLK1 in the regulation of global DNA methylation, we first treated DU145 or BT549 cells with BI6727, a highly specific PLK1 inhibitor, and examined the status of global DNA methylation by DNA dot blot using anti-5mC antibody. Beyond our expectations, BI6727 dramatically decreased the levels of global DNA methylation in a dose-dependent manner (Fig. 1A). Consistent with the data of DNA dot blot, ELISA assays validated that BI6727 substantially reduced the levels of global DNA methylation (Fig. 1B). In parallel, we depleted PLK1 with siRNA in DU145 and BT549 cells (Fig. 1C) and monitored the levels of global DNA methylation. Consistent with the BI6727 data, PLK1 depletion with siRNA significantly reduced the global DNA methylation level (Fig. 1D–F). Since most of the DNA methylation occurs on the newly replicated strand, we examined the impact of PLK1 deletion on DNA methylation during S phase. The data showed that PLK1 depletion significantly decreased the level of global DNA methylation in S phase (Supplementary Fig. S1). These data suggested that PLK1 plays a critical role in the maintenance of global DNA methylation.

### PLK1 inhibition increased the expression level of TSGs

**Fig. 2:**
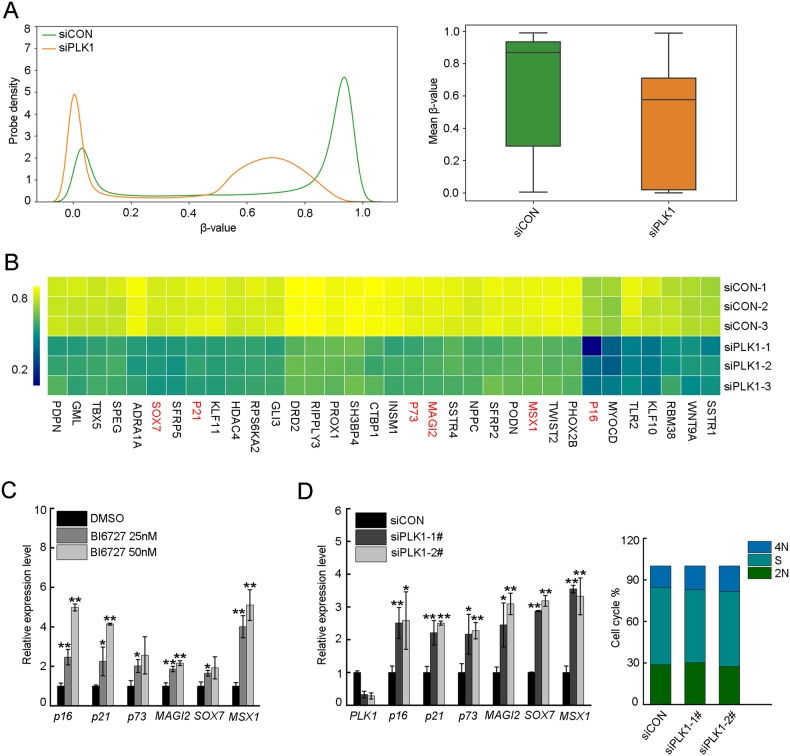
PLK1 inhibition increased the expression level of tumor suppressor genes. **A** DU145 cells were transfected with PLK1 siRNA, and the genomic DNA was isolated. The global DNA methylation profiling was performed by Illumina Infinium Methylation EPIC arrays. All array probes for each group were used to generate density plots (left) and boxplots (right). In density plots, X-axis indicates β value; The scores in the range between 0 and 1 indicate the level of DNA methylation. Y axis indicates the probability densities which describe the distribution of β values for all probes. Whisker boxplots (right) represent the 25th–75th percentiles, with midlines indicating the median values. Whiskers extend to the minimum/maximum values. **B** Heatmaps shows β values of probe in the promoter regions of tumor suppressor genes. The TSGs marked in red were selected for further validation. **C** DU145 were treated with DMSO or different doses of BI6727 for 48 h. The mRNA levels of tumor suppressor genes were analyzed by RT-PCR, *ACTIN* was used as an internal control. **D** DU145 cells were transfected with scrambled or two different PLK1 siRNAs, and then cell cycle was synchronized at S phase. The cell cycle phase distribution was analyzed by flow cytometry. The mRNA levels of tumor suppressor genes were analyzed by RT-PCR, *ACTIN* was used as an internal control. Data were expressed as mean ± SD from three separate experiments, and were analyzed for statistical significance *P < 0.05, **P < 0.01.

To further validate the roles of PLK1 in the regulation of DNA methylation, we analyzed DNA methylation landscape by using Illumina Infinium Methylation EPIC arrays when PLK1 was depleted with siRNA in DU145 cells. As expected, PLK1 depletion significantly decreased the level of genome-wide DNA methylation (Fig. 2A). Aberrant DNA hypermethylation-induced silencing of TSGs is one fundamental hallmarks of cancer. We found that PLK1 was positively correlated with the DNA methylation levels of TSGs promoter region (Fig. 2B). Further quantitative analysis showed that PLK1 inhibition significantly elevated the mRNA and protein level of TSGs (Fig. 2C, D and Supplementary Fig. S2). These data suggested that PLK1 suppressed the transcription of TSGs by elevating the level of DNA methylation in the promoter regions of TSGs.

### PLK1 promotes DNMT1 recruitment to chromatin by sustaining UHRF1 protein stability

**Fig. 3:**
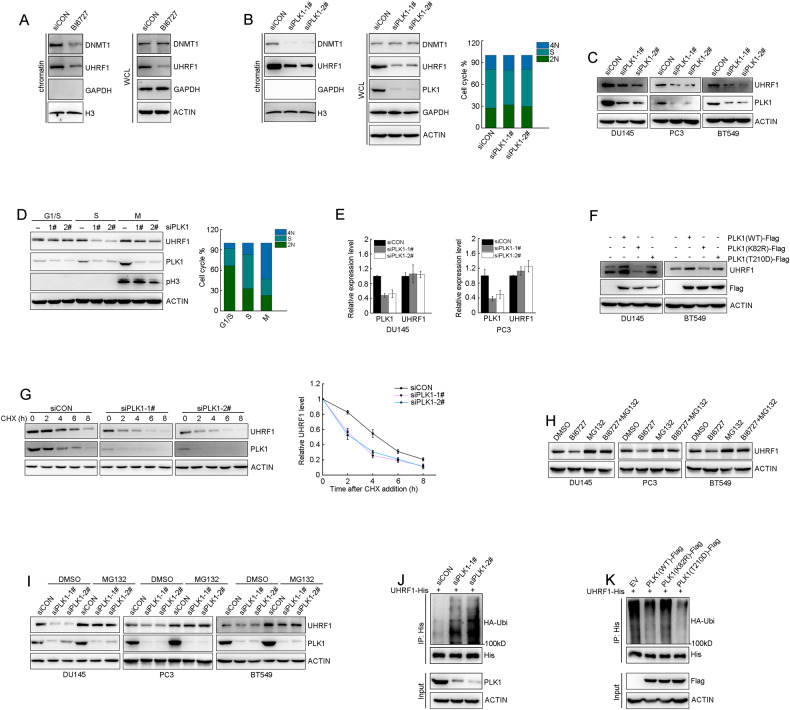
PLK1 promotes DNMT1 binding to chromatin by sustaining UHRF1’s protein stability. **A** DU145 cells were treated with DMSO or BI6727 (50 nM) for 48 h, and the levels of chromatin-binding UHRF1 and DNMT1 proteins or whole-cell lysates (WCL) were assessed by immunoblotting. **B** DU145 cells were transfected with scrambled or two different PLK1 siRNA, and then cell cycle was synchronized in S phase. The cell cycle phase distribution was analyzed by flow cytometry. The levels of chromatin-binding UHRF1 or DNMT1 protein were assessed by immunoblotting. **C** Cells was transfected with scrambled or two different PLK1 siRNAs for 72 h. The protein levels of PLK1 and UHRF1 were assessed by immunoblotting. **D** DU145 cells were transfected with scrambled or two different PLK1 siRNAs. The cells were synchronized in the indicated cell cycle phase and cell cycle phase distribution was analyzed by flow cytometry. The protein levels of PLK1 and UHRF1 were assessed by immunoblotting. **E** Cell was transfected with scrambled or two different PLK1 siRNAs for 48 h. The mRNA levels of *PLK1* or *UHRF1* genes were analyzed by RT-PCR. *ACTIN* was used as an internal control. **F** Cell was transfected with the plasmids expressing PLK1(WT), PLK1(K82R), or PLK1(T210D) as indicated for 72 h. The protein levels of PLK1 and UHRF1 were assessed by immunoblotting. **G** PLK1-depleted DU145 cells were treated with cycloheximide (CHX, 50 μg/ml) for the indicated times, and the endogenous UHRF1 protein levels were monitored by immunoblotting. The protein levels were quantified by grayscale analysis. The data are shown as the mean ± SD from three separate experiments. **H** Cell was treated with BI6727 (50 nM) for 18 h, followed by 50 μM MG132 for 6 h. UHRF1 protein was assessed by immunoblotting. **I** Cell was transfected with scrambled or PLK1 siRNAs for 72 h. Cell was treated with 50 μM MG132 for 6 h, and the protein levels of PLK1 and UHRF1 were assessed by immunoblotting. **J** The plasmids expressing HA-ubiquitin and UHRF1-His together with PLK1 siRNAs were transfected to cell for 48 h and then treated with 50 μM MG132 for 6 h. UHRF1 protein was immunoprecipitated with anti-His antibody and the polyubiquitylated UHRF1 was assessed with anti-HA antibody. **K** The plasmids expressing PLK1(WT)-Flag, PLK1(K82R)-Flag, or PLK1(T210D)-Flag together with UHRF1-His and HA-ubiquitin were co-transfected to cell for 48 h and then treated with 50 μM MG132 for 6 h. UHRF1 protein was immunoprecipitated with anti-His antibody, and the polyubiquitylated UHRF1 was assessed with anti-HA antibody.

Previous studies reported that UHRF1 binds to the hemi-methylated CpG, and recruits DNMT1 to ensure faithful propagation of DNA methylation and maintain the global DNA methylation [16, 25]. We tested whether PLK1 controls global DNA methylation through the UHRF1-DNMT1 pathway. DU145 cells were treated with PLK1 inhibitor BI6727, and the levels of chromatin-bound UHRF1 or DNMT1 were assessed. As shown in Fig. 3A, BI6727 remarkably decreased the binding degree of UHRF1 and DNMT1 proteins to chromatin. Consistent with the BI6727 data, PLK1 depletion significantly reduced the binding of UHRF1 or DNMT1 to chromatin in S phase (Fig. 3B). The results demonstrated that PLK1 is required for UHRF1 and DNMT1 protein binding to chromatin.

BI6727 or PLK1 depletion dramatically decreased UHRF1 protein levels, but had no impact on DNMT1 (Fig. 3A–C). The data suggested that PLK1 controls DNA methylation through UHRF1-mediated DNMT1 protein binding to chromatin. Since the protein level of PLK1 is coordinately regulated during cell cycle progression, we evaluated the impact of PLK1 depletion on UHRF1 protein in different cell cycle phases. The data showed that PLK1 depletion significantly decreased UHRF1 protein level in S phases but only moderately decreased that in M phases (Fig. 3D). We excluded the impact of PLK1 depletion on the mRNA level of UHRF1 by RT-PCR (Fig. 3E). We treated DU145 and PC3 cells with BI6727, and assessed the protein and mRNA levels of UHRF1. Consistently, BI6727 dramatically induced UHRF1 protein degradation, but did not change mRNA levels (Supplementary Fig. S3A and S3B). The results indicated that PLK1 sustains UHRF1 expression through a post-translational mechanism. To further clarify whether PLK1 regulates UHRF1 protein stability depending on its kinase activity, we constructed a constitutively active PLK1 mutant (PLK1-T210D) and a kinase-dead mutant (PLK1-K82R). The ectopic expression of wild-type PLK1 (PLK1-WT) and PLK1-T210D significantly elevated the levels of endogenous UHRF1 protein, while PLK1-K82R did not elevate, but moderately decreased UHRF1 protein level (Fig. 3F). These results demonstrated that UHRF1 protein stability is dependent on kinase activity of PLK1. To further validate these results, we assessed the half-life of UHRF1 protein when PLK1 was depleted in DU145 cells. The protein synthesis was inhibited by cycloheximide (CHX), and UHRF1 protein stability was assessed at continuous time points. PLK1 depletion with siRNA accelerated UHRF1 protein degradation and shortened the half-life of UHRF1 protein (Fig. 3G). Consistently, BI6727 significantly shortened the half-life of endogenous UHRF1 protein in DU145 and BT549 cells (Supplementary Figs. S4A and S4B). Taken together, the results indicated that PLK1 sustained UHRF1 protein stability by inducing UHRF1 phosphorylation.

To determine how PLK1 modulates UHRF1 protein stability, we treated cells with BI6727 and MG132, a specific proteasome inhibitor. UHRF1 degradation protein was reversed by MG132, indicating that PLK1 sustained UHRF1 protein stability through the ubiquitination-proteasome pathway (Fig. 3H). Consistently, PLK1 depletion with siRNA induced the degradation of endogenous UHRF1 protein in DU145, PC3 and BT549 cells, and the protein degradation was reversed by MG132 (Fig. 3I). The results were further validated by the in vivo ubiquitination assay. The plasmids encoding HA-ubiquitin or UHRF1-His were co-transfected into HEK293T cells. UHRF1 protein was immunoprecipitated with anti-His antibody, and the ubiquitinated protein was detected with HA antibody. The knockdown of PLK1 remarkably elevated the ubiquitination level of UHRF1 protein (Fig. 3J). Since PLK1 is a protein kinase, we next tested whether the kinase activity of PLK1 is required for the polyubiquitination of UHRF1 protein. Comparing to PLK1-WT or PLK1-T210D, the kinase-dead mutant PLK1-K82R significantly increased the in vivo UHRF1 polyubiquitination level (Fig. 3K). These results indicated that kinase activity of PLK1 is required for the maintenance of UHRF1 protein stability.

### PLK1 physically interacts with UHRF1 protein

**Fig. 4:**
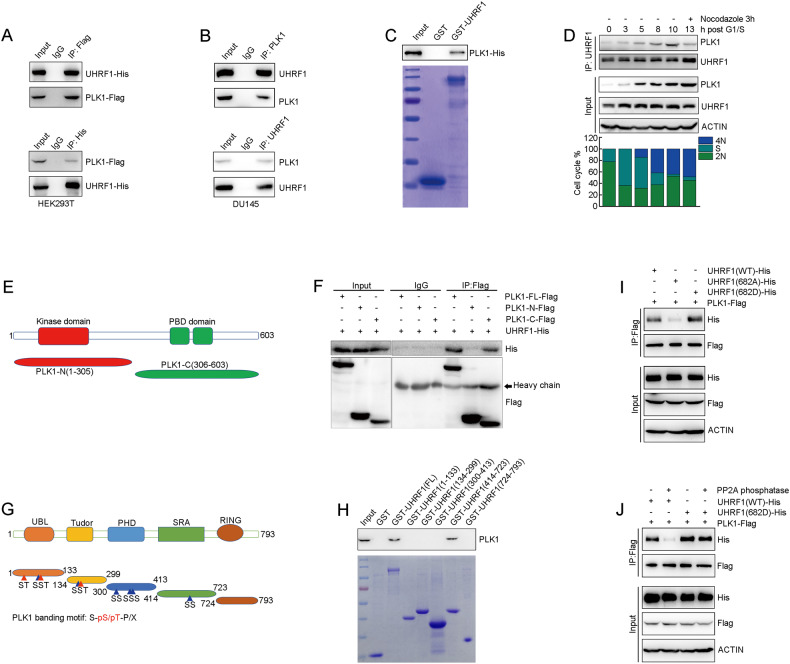
PLK1 interacts with UHRF1. **A** HEK293T cells were co-transfected with UHRF1-His and PLK1-Flag plasmids. PLK1 or UHRF1 was immunoprecipitated with anti-Flag or anti-His antibody, and UHRF1 or PLK1 was assessed with anti-His or anti-Flag antibody by immunoblotting. **B** Cell lysates were subjected to immunoprecipitation with anti-PLK1 or anti-UHRF1 antibody. The immunoprecipitated protein was then blotted with the indicated antibodies. **C** The recombinant GST, GST-UHRF1 and PLK1-His proteins were purified and incubated in vitro as indicated. The protein interaction between UHRF1 and PLK1 was then examined by GST-pull down assay. **D** DU145 cells were synchronized at G1/S phase by double-thymidine blocking, and then released to cell cycle with fresh medium. The cells were arrested at M phase with Nocodazole. Whole cell lysates were immunoprecipitated with anti-UHRF1 antibody, and PLK1 was assessed as indicated. Cell cycle phase distribution was analyzed by flow cytometry. **E** Schematic diagram of PLK1 protein domains. **F** HEK293T cells were transfected with UHRF1-His and Flag-tagged plasmids expressing PLK1 full length or its truncated mutants as indicated. Cell lysates were immunoprecipitated with anti-Flag antibody, and UHRF1 was analyzed by western blot. **G** Schematic diagram of UHRF1 protein domains. The blue triangle represents the SS amino acid site and the red triangle represents the ST amino acid site. **H** In vitro GST pull-down assays were performed by using various GST-fused UHRF1 proteins immobilized on glutathione beads. Various immobilized GST-UHRF1 proteins were incubated with DU145 cell lysates, and PLK1 was analyzed by western blot. **I** DU145 cells were transfected with plasmids expressing PLK1-Flag and UHRF1-His wild type or mutants as indicated. PLK1 was immunoprecipitated with anti-Flag antibody, and UHRF1 was assessed with anti-His antibody by western blotting. **J** DU145 cells were transfected with plasmids expressing PLK1-Flag and UHRF1-His wild type or mutants as indicated, and then cell cycle was synchronized at S phase. The cell lysates were separated into two parts, individually treated with or without PP2A phosphatase. PLK1 was immunoprecipitated with anti-Flag antibody, and UHRF1 was assessed with anti-His antibody by western blotting.

It is well known that physical protein interaction is required for PLK1-induced UHRF1 phosphorylation. We sought to detect the protein interaction of PLK1 and UHRF1. In our previous study, we screened PLK1-interactive proteins by mass spectrometry (data not shown) and for the first time found that UHRF1 is a PLK1-interactive protein. The protein interaction between UHRF1 and PLK1 was further validated by co-immunoprecipitation. PLK1 or UHRF1 was individually fused with Flag or His tags, and both were co-transfected to HEK293T cells. PLK1 or UHRF1 protein was immunoprecipitated with anti-Flag or anti-His antibody, and UHRF1 or PLK1 protein was detected in the immunoprecipitated protein complex (Fig. 4A). The endogenous UHRF1 and PLK1 protein interaction was validated by reciprocal co-immunoprecipitation in DU145 and BT549 cells. UHRF1 protein was detected when PLK1 was immunoprecipitated, and conversely PLK1 was detected when UHRF1 was immunoprecipitated (Fig. 4B and Supplementary Fig. S5A). Additionally, the physical interaction of UHRF1 and PLK1 proteins was further confirmed by GST-pull down assay (Fig. 4C and Supplementary Fig. S5B). To monitor the strength of UHRF1 and PLK1 protein interaction at different cell cycle phases, we enriched DU145 cells at different phases with thymidine and nocodazole treatment. The protein interaction of UHRF1 and PLK1 gradually increased when the cell cycle was released from the G1/S phase, but remarkably decreased at M phase (Fig. 4D).

PLK1 has a kinase domain (KD) at the amino terminal and two polo-box domains (PBDs) at the carboxyl terminal (Fig. 4E). We constructed two plasmids individually encoding the N-terminal fragment containing the kinase domain, or the C-terminal fragment containing a PBD domain of PLK1. Using co-transfection and co-immunoprecipitation, we identified that UHRF1 interacts with the PBD regions of PLK1 (Fig. 4F). To identify the domain of UHRF1 protein responsible for PLK1 binding, we constructed a series of truncations of UHRF1 in GST vector (UBL domain-containing fragment 1-133aa; TTD domain-containing fragment 134-299aa; PHD domain-containing fragment 300-413aa; SRA domain-containing fragment 414-723aa; RING domain-containing fragment 724-793aa) (Fig. 4G). GST pull-down assays were performed by co-incubating the recombinant UHRF1 truncated proteins with cell lysates. The data demonstrated that PLK1 interacted with the SRA domain of UHRF1 protein (Fig. 4H). PBD is a phosphopeptide-binding domain required for the interaction of PLK1 with substrate that has undergone pre-phosphorylation at specific docking sites [30, 31]. The PBD domain of PLK1 protein is well known to recognize a consensus sequence of S-pS/pT-P/X [31]. After carefully analyzing the sequence of the SRA domain-containing fragment of UHRF1 protein, we identified one potential PBD-binding site, Ser682 (Fig. 4G). To determine whether phosphorylation of UHRF1 at serine 682 creates a docking site allowing subsequent binding to the PBD of PLK1, we mutated Ser682 to Ala or Asp, followed by co-immunoprecipitation. The data showed that the binding strength of UHRF1 to PLK protein significantly reduced when UHRF1 Ser682 was mutated (S682A) compared to WT and its mimic mutant (S682D) (Fig. 4I). A GST-pull down assay revealed that Ser682 of UHRF1 was required for the physical interaction of UHRF1 with PLK1 (Supplementary Fig. S6). Furthermore, DU145 cells were transfected with plasmids expressing PLK1-Flag and UHRF1-His wild type or its mimic mutant (S682D), and then treated with PP2A phosphatase before immunoprecipitation. The data showed that PP2A phosphatase significantly abolished the protein interaction of PLK1 and UHRF1 wild type, but had little impact on the interaction of PLK1 and UHRF1 mimic mutant (S682D) (Fig. 4J). The results demonstrated that the Ser682 phosphorylation of UHRF1 is required for subsequent protein interaction with PLK1.

### PLK1 phosphorylates UHRF1 at ser265 to sustain UHRF1 protein stability

**Fig. 5:**
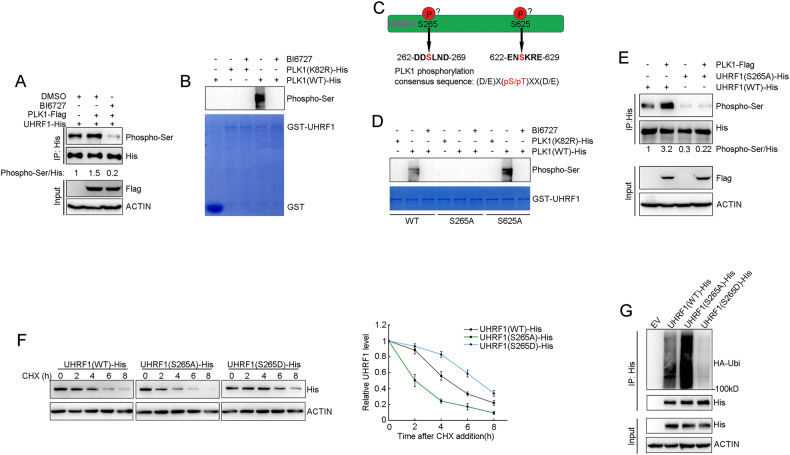
PLK1 promotes UHRF1 protein stability by phosphorylating Ser265. **A** DU145 cells were transfected with plasmids expressing PLK1-Flag and UHRF1-His for 60 h, then treated with BI6727 (50 nM) for 12 h. UHRF1 was immunoprecipitated with anti-His antibody, and the total phosphorylation level was measured with anti-Phospho-Ser antibody. Protein levels were quantified by grayscale analysis. **B** Bacterial-expressed GST and GST-UHRF1 were subjected to in vitro kinase assay. BI6727, wild-type PLK1 (active form) and K82R (inactive form) were used in the kinase assays, and the phosphorylation level of UHRF1 was assessed with anti-phosphoserine antibody. The loading controls are shown in the bottom panel (stained by Coomassie blue). **C** Schematic diagram of putative PLK1-mediated phosphorylation sites in UHRF1 protein. **D** Bacterial-expressed GST-UHRF1 wild type or mutants were subjected to in vitro kinase assay. BI6727, wild-type PLK1 (active form) and K82R (inactive form) were used in the kinase assays, and the phosphorylation level of UHRF1 was assessed with anti-phosphoserine antibody. The loading controls are shown in the bottom panel (stained by Coomassie blue). **E** DU145 cells were transfected with plasmids expressing PLK1-Flag and UHRF1-His wild type and mutants, and then cell cycle was synchronized in S phase. UHRF1 was immunoprecipitated with anti-His antibody, and the total phosphorylation level was measured with anti-Phospho-Ser antibody. Protein levels were quantified by grayscale analysis. **F** HEK-293T cells were transfected with the plasmids expressing UHRF1(WT)-His, UHRF1(S265A)-His or UHRF1(S265D)-His for 60 h, and then were treated with CHX (50 μg/ml). The cells were harvested at the indicated time points. UHRF1 protein were analyzed by immunoblotting, and were quantified by grayscale analysis. The data are expressed as mean ± SD. **G** HEK-293T cells stably expressing HA-ubiquitin were transfected with the indicated plasmids for 48 h, and then treated with 50 μM MG132 for 6 h. UHRF1 was immunoprecipitated with anti-His antibody, and the polyubiquitylated UHRF1 protein was detected by anti-HA antibody.

To validate whether UHRF1 is a phosphorylation substrate of PLK1, we co-transfected plasmids encoding UHRF1-His and PLK1-Flag to DU145 cells, which were then treated with BI6727. UHRF1 was first immunoprecipitated with anti-His antibody, and the phosphorylation level of UHRF1 was assessed using an anti-pan-phospho-Ser antibody. PLK1 over-expression elevated UHRF1 phosphorylation levels, but BI6727 impaired PLK1-elevated UHRF1 phosphorylation (Fig. 5A). Furthermore, we performed the in vitro kinase assays to validate whether UHRF1 is directly phosphorylated by PLK1. In comparison to the kinase-inactive mutation (PLK1-K82R), wild-type (PLK1-WT) could to phosphorylate UHRF1 in vitro, but the BI6727 abolished UHRF1 phosphorylation by wild-type (PLK1-WT) (Fig. 5B). The results revealed that UHRF1 is a phosphorylation substrate of PLK1. Further studies identified Ser265 and Ser625 as the potential PLK1 phosphorylation sites, which closely resemble the consensus substrate sequence for PLK1 ([D/E]-X[pS/pT]-X-X-[D/E]) [32] (Fig. 5C). We then individually mutated these two residues to Ala, and confirmed the bona fide PLK1 phosphorylation sites on UHRF1 by using in vitro kinase assays. The results showed that in comparison to wild-type UHRF1 (UHRF1-WT), the mutations to Ala of Ser265, but not Ser625, inhibited the phosphorylation of UHRF1 by PLK1. These data validated Ser265 of UHRF1 as an authentic phosphorylation site of PLK1 kinase (Fig. 5D). Furthermore, we co-transfected plasmids encoding PLK1-Flag and UHRF1-His wild type or its mutant (S265A) to DU145 cells, and then the cells were synchronized at S phase. UHRF1 was immunoprecipitated with anti-His antibody, and the phosphorylation level of UHRF1 was assessed using an anti-pan-phospho-Ser antibody. Compared to UHRF1 wild type, the phosphorylation level of UHRF1 mutant (S265A) was significantly decreased. More importantly, PLK1 over-expression didn’t elevate the phosphorylation of the mutant (S265A) compared to UHRF1 wild-type. These data validated Ser265 of UHRF1 was the unique phosphorylation site of PLK1 kinase (Fig. 5E).

To evaluate the impact of PLK1 phosphorylation on UHRF1 protein stability, we ectopically elevated the expression of wild-type or mutated UHRF1, and assessed the half-life of UHRF1 protein. In comparison to wild-type UHRF1, mutations of Ser265 to Ala (S265A, dead mutation) significantly shortened the half-life of UHRF1 protein, and conversely the mutation of Ser265 to Asp (S265D, mimic mutation) remarkably prolonged the half-life of UHRF1 protein (Fig. 5F). Furthermore, S265A significantly promoted the polyubiquitination levels of UHRF1 protein compared to WT and S265D (Fig. 5G). Altogether, these data demonstrated that PLK1, by phosphorylating the Ser265 site, reduced the ubiquitination levels of UHRF1 protein, thereby sustaining its protein stability.

### PLK1 enhanced the protein interaction of UHRF1 and USP7

**Fig. 6:**
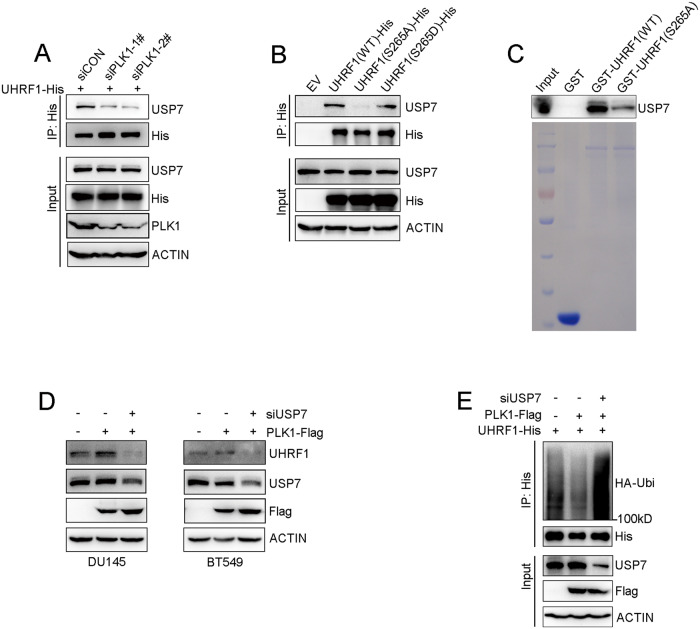
PLK1 sustaining the protein interaction of UHRF1 and USP7 by induces UHRF1 phosphorylation at Ser265. **A** DU145 was co-transfected with the indicated plasmids and siRNAs for 72 h, UHRF1 was immunoprecipitated with anti-His antibody, and USP7 was analyzed by immunoblotting. **B** DU145 cells were transfected with the plasmids expressing UHRF1(WT)-His, UHRF1(S265A)-His, or UHRF1(S265D)-His mutants as indicated for 72 h. UHRF1 was immunoprecipitated with anti-His antibody, and USP7 was assessed by western blot. **C** In vitro GST pull-down assays were performed using GST-UHRF1 wild type or mutants as indicated, and immobilized on glutathione beads. Various immobilized GST-UHRF1 proteins were incubated with cell lysates of DU145, and USP7 was assessed by western blot. **D** DU145 and BT549 cells were co-transfected with the indicated plasmids and siRNAs for 72 h, and UHRF1 and USP7 were analyzed by western blot. **E** Plasmids expressing UHRF1-His and HA-ubiquitin, together with the indicated plasmids and siRNAs, were co-transfected to cell for 48 h, and then treated with 50 μM MG132 for 6 h. UHRF1 was immunoprecipitated with anti-His antibody, and the polyubiquitylated UHRF1 protein was detected by anti-HA antibody.

We further explored the mechanism by which PLK1 reduced UHRF1 protein ubiquitination levels. It has been reported that USP7 is a deubiquitinase releasing the ubiquitylation status of UHRF1 protein, and sustaining protein stability [1, 33]. Therefore, we hypothesized that PLK1-regulated phosphorylation is required to sustain the physical interaction of UHRF1 and USP7. To address this hypothesis, we assessed the impact of PLK1 on the protein interaction of UHRF1 and USP7 by immunoprecipitation. Knockdown of PLK1 with siRNAs markedly reduced the protein interaction of UHRF1 and USP7 (Fig. 6A). The protein interaction of UHRF1 and USP7 significantly decreased when the phosphorylation site S265 was mutated (S265A) compared to WT and its mimic mutant (S265D) (Fig. 6B). GST-pull down assay validated that S265A mutation significantly decreased the protein interaction of UHRF1 and USP7 (Fig. 6C). These results suggested that PLK1 phosphorylation on UHRF1 is required for the protein interaction of UHRF1 and USP7. It has been reported that CDK1 induced UHRF1 phosphorylation at S652 at M phase, which inhibited its physical interaction with the deubiquitinase USP7 [1]. Here, we investigated the impact of CDK1-induced UHRF1 phosphorylation on the protein interaction between UHRF1 and PLK1. Since the UHRF1 transcript used in our study lacks 13 amino acids in the UBL domain compared to that in a previous publication [1], the site S652 of UHRF1 in that publication becomes the site S639 in our study. As expected, the protein binding strength of UHRF1 and PLK1 significantly reduced when UHRF1 was phosphorylated by CDK1 (Supplementary Fig S7). To further verify whether PLK1 sustains the protein stability of UHRF1 was dependent on USP7, we depleted USP7 with siRNA and artificially elevated PLK1 before UHRF1 protein stability was assessed. The results showed that PLK1 increased UHRF1 protein levels, while USP7 depletion induced UHRF1 protein degradation even though PLK1 was artificially elevated (Fig. 6D). Furthermore, PLK1 decreased the ubiquitination level of UHRF1 protein, while USP7 depletion rescued the ubiquitination level of UHRF1 protein (Fig. 6E). These data indicated that PLK1 phosphorylation promoted the protein stability of UHRF1 by enhancing the physical interaction of UHRF1 and USP7.

### Phosphorylation of UHRF1 at Ser265 was required for sustaining cell viability

**Fig. 7:**
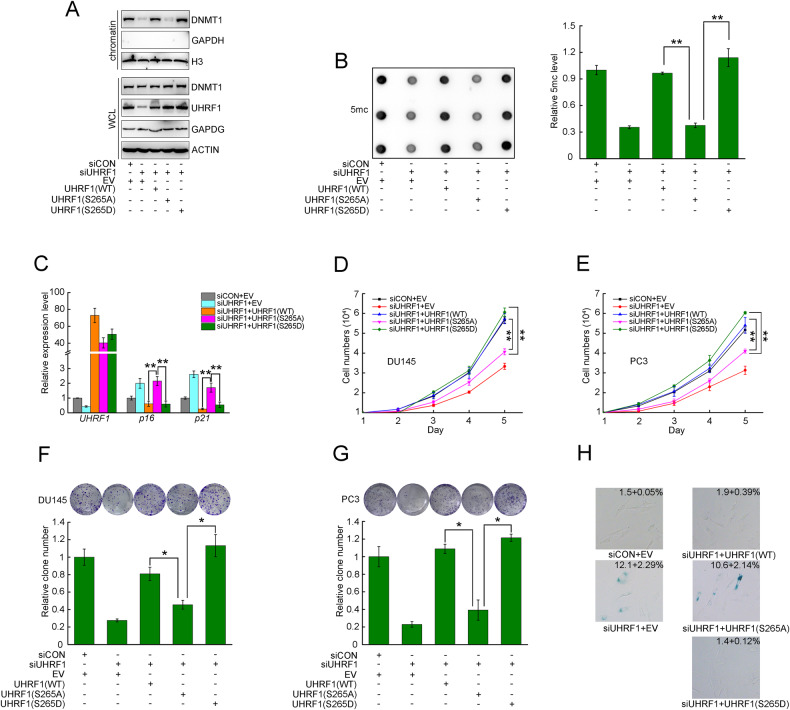
Phosphorylation of UHRF1 was essential for sustaining cell viability. **A**, **B** DU145 cells were co-transfected with the indicated plasmids and siRNAs, and cell cycle was synchronized at S phase. The levels of chromatin-binding DNMT1 proteins and global DNA methylation were assessed. **C** DU145 cells were co-transfected with the indicated plasmids and siRNAs for 72 h. The *UHRF1*, *p16* and *p21* genes were analyzed by RT-PCR. *ACTIN* was used as an internal control. **D**, **E** DU145 and PC3 cells were co-transfected with the indicated plasmids and siRNAs. They were seeded at 1 × 10^4^ cells in triplicate 60 mm plates. Cells were trypsinized and counted at indicated time points. Data represent the mean ± SD of each group from three separate experiments. **P < 0.01. **F**, **G** Colony formation assay using DU145 and PC3 UHRF1 cells were co-transfected with the indicated plasmids and siRNAs. Data represent the mean ± SD of each group from three separate experiments. *P < 0.05. **H** HDF cells were co-transfected every 3 days with the indicated plasmids and siRNAs, senescence-associated β-galactosidase (SA-β-gal) staining was performed after 6 days of co-transfected. Data represent the mean ± SD of SA-β-gal-positive cells each group from three separate experiments.

UHRF1 establishes and maintains DNA methylation patterns in mammalian cells by recruiting DNMT1. We further validated whether PLK1-induced UHRF1 phosphorylation is required for DNMT1 binding to chromatin and maintaining global DNA methylation. We co-transfected UHRF1 siRNAs and plasmids encoding UHRF1 WT or mutants to DU145 cells, and the cells were synchronized at S phase. We assessed the levels of DNMT1 binding to chromatin, as well as the levels of global DNA methylation. Compared to UHRF1 WT or S265D, the dead mutation(S265A) significantly decreased the chromatin-binding levels of DNMT1 and global DNA methylation (Fig. 7A, B).

It was reported that UHRF1 depletion remarkably decreased cell viability by elevating the expression of TSGs in several cancer types [27]. We further examined the effect of UHRF1 phosphorylation on p21 and p16 levels. Compared to UHRF1 WT or S265D, the dead mutation (S265A) significantly elevated the mRNA and protein levels of p16 and p21 (Fig. 7C and Supplementary Fig S8). As key TSGs and cell cycle inhibitors, p16 or p21 significantly inhibited cell growth and induced cellular senescence [28]. The dead mutation (S265A) of UHRF1 remarkably inhibited cell proliferation and decreased cell viability compared to UHRF1 WT and S265D (Fig. 7D–G). Furthermore, we depleted UHRF1 in human fibroblasts, and then re-expressed UHRF1 WT and mutants back by transient transfection. The data showed that UHRF1 WT and S265D, rather than S265A rescued cellular senescence (Fig. 7H). These data indicated that PLK1-induced UHRF1 phosphorylation is required for the maintenance of cell viability by suppressing the expression of TSGs through UHRF1-DNMT1-regulated DNA methylation.

## Discussion

PLK1 is a member of the serine/threonine kinase family and a key mitotic regulator that controls centrosome maturation, mitosis entrance, chromosome segregation and cytokinesis [34, 35]. Additionally, PLK1 regulates DNA replication during cell cycle S phase [36]. Previous studies demonstrated that DNA replication is accompanied by DNA methylation [16]. In the present study, by using such different DNA methylation analysis approaches as ELISA, Illumina Arrays, DNA dot blot and HPLC, we for the first time reported that PLK1 plays a critical role in the regulation of genome-wide DNA methylation (Fig. 1).

By inducing the phosphorylation of substrates, PLK1 plays critical roles in various types of diseases including cancer. Several PLK1 substrates have been identified, including NOTCH1, FOXO1, and FOXM1 [37–39]. we identified UHRF1 as a novel PLK1 substrate in the present study (Fig. 4). PLK1 induced UHRF1 phosphorylation via the residue Ser265, and sustained the protein stability of UHRF1 by decreasing the in vivo level of UHRF1 polyubiquitination (Fig. 5). USP7 is a critical deubiquitinase for the maintenance of UHRF1 protein stability [1]. PLK1 knockdown with siRNAs markedly reduced the protein interaction of UHRF1 and USP7. The phosphorylation of UHRF1 at residue Ser265 by PLK1 is a prerequisite for the interaction of UHRF1 and USP7. USP7 knockdown significantly decreased the protein level of UHRF1 even when PLK1 was artificially elevated (Fig. 6). These results unambiguously demonstrated that PLK1-induced UHRF1 phosphorylation is crucial for USP7-maintained UHRF1 protein stability.

The recruitment of DNMT1 to chromatin is required to maintain DNA methylation levels [40]. In the present study, PLK1 inhibition markedly reduced the level of chromatin-bound UHRF1 and DNMT1 proteins (Fig. 3A, B). UHRF1, as an essential accessory factor, recruits DNMT1 to the DNA replication fork, and preferentially converts the hemi-methylated CpGs to fully methylated status during DNA replication [41, 42]. In the present study, PLK1 inhibition significantly decreased global DNA methylation levels (Fig. 1), which is similar to the effects of UHRF1 knockdown. PLK1 inhibition significantly induced UHRF1 protein degradation, and further reduced DNMT1 recruitment to chromatin (Fig. 3). The results suggest that PLK1 promotes DNMT1 recruitment to chromatin, especially to the newly replicated DNA in S phase, by sustaining UHRF1 protein stability in a kinase-dependent manner. Because of the protein interaction between PLK1 and UHRF1 occurs in different cell cycles (Fig. 4D), DNA methylation may not be the only biological consequence of PLK1-induced UHRF1 phosphorylation. In addition to cell proliferation and DNA damage repair, UHRF1 has been reported as a key molecule in suppressing the expression of TSGs [27]. In the present study, PLK1 inhibition significantly elevated the expression level of such TSGs as *p16*, *p21*, *p73*, *MAGI2*, *SOX7* and *MSX1* (Fig. 2C, D). Moreover, we further validated that PLK1-induced UHRF1 phosphorylation and protein stability is required for the silencing of TSGs and sustaining cell viability for cancer cells (Fig. 7). UHRF1 as an adapter protein, bridges DNA methylation and histone modifications, and suppresses the expression of TSGs by assembling a complex including histone modifiers and DNMT1 [43]. However, it deserves further investigations whether PLK1-induced UHRF1 phosphorylation is required for the recruitment of histone modifiers.

**Fig. 8:**
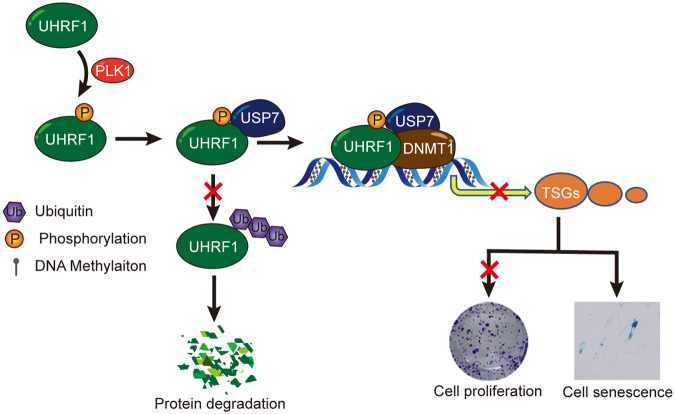
Working model showing that PLK1 enhances UHRF1 interaction with USP7 by phosphorylation and sustains the protein stability of UHRF1. UHRF1 promotes DNMT1 binding to chromatin and maintenance DNA methylation, thereby promoted cell proliferation and inhibited cell senescence through inhibits the expression of TSGs.

Altogether, we for the first time identified the key roles of PLK1 in the maintenance of DNA methylation through the UHRF1-DNMT1 pathway. PLK1 induced UHRF1 phosphorylation and sustained its protein stability by recruiting a deubiquitinase USP7. Reversely, PLK1 knockdown with siRNA or specific kinase inhibitor BI6727 significantly abolished the protein interaction of UHRF1 and USP7, thereby accelerating UHRF1 protein degradation through the ubiquitin-proteasome pathway. UHRF1 protein degradation decreased the recruitment of DNMT1 to the chromatin, and reduced the level of global DNA methylation and elevated the gene expression of TSGs, thereby inhibiting cell proliferation and inducing cellular senescence (Fig. 8). These results suggest that the combination of PLK1 inhibitor with DNMT1 inhibitor may exert significant synergistic efficacy for future cancer therapy.

## Materials and methods

### Cell culture and cell cycle synchronization

DU145, PC3, BT549 and HEK293T cells were purchased from the National Collection of Authenticated Cell Cultures. Human dermal fibroblast (HDF) cells were gifted by Center for Molecular Medicine of Xiangya Hospital, Central South University. These cells were maintained in DMEM or 1640 medium supplemented with 10% fetal bovine serum (FBS), 1% penicillin and streptomycin. For synchronization in G1/S phase, cells were treated with 2 mM thymidine (Sigma-Aldrich, Shanghai) for 18 h, and then washed and cultured in fresh medium for 9 h. The 18 h thymidine treatment was repeated once. For synchronization in S phase, DU145 cells first were synchronized at G1/S phase using a double treatment of thymidine, and then were washed and cultured in fresh medium for 3 h. For synchronization in mitotic phase, DU145 cells were treated with nocodazole (50 ng/ml) (Chemicals Selleck, Selleck, Shanghai) for 16 h.

### Plasmids and siRNAs

The cDNA fragment of *PLK1* or *UHRF1* gene was generated by PCR, and then subcloned into the individual vectors to construct pCMV-PLK1-Flag, pET28a-PLK1, pGEX-4T-1-PLK1, pCDNA3.1-UHRF1-His, and pGEX-4T-1-UHRF1 plasmids. All mutants were constructed by site-directed mutagenesis and verified by DNA sequencing. siRNA transfection was performed with siRNA duplexes against PLK1 and UHRF1 using a Mirus Transfection Kit according to the manufacturer’s instructions (Mirus, Madison, WA). The siRNA sequences used for PLK1 knockdown were (5’-3’): PLK1#1 (CGAUACUACCUACGGCAAA), PLK1#2 (GGGCGGCUUUGCCAAGUGC). The siRNA sequences used for UHRF1 knockdown were (5’-3’): GCCUUUGAUUCGUUCCUUC.

### RNA extraction and real-time quantitative PCR

The total RNA was extracted following the RNAiso Plus manufacturer’s protocol (Takara Bio.Beijing, China). The RNA concentration and quality were determined by spectrophotometer before its conversion to cDNA. The mRNA level of genes was measured by an Applied Biosystems 7500 Real-time PCR system (Thermo Fisher Scientific, China) according to the manufacturer’s instructions. Primers for RT-PCR were listed in Table S1.

### Antibodies and chemicals

The antibodies and chemicals used in the study were anti-Flag (Sigma, F1804 and Cell Signal Technology, CST 14793), anti-His (Genscript, A00186), anti-phosphoseine (Santa Cruz Biotechnology, Santa sc-81516), anti-PLK1 (Cell Signal Technology, CST 4513 and Santa, sc-17783), anti-UHRF1 (Proteintech, 21402-1-AP and Santa, sc-373750), anti-DNMT1 (Abclonal, A5495), anti-p21 (CST, 2947), anti-p16 (CST, 18769), anti-HA (CST, 3724), anti-5-Methylcytosine (CST, 28692), anti-Phospho-Histone H3 (Ser 10) (Proteintech, 66863-1) and anti-β-actin (Abclonal, AC004). Cycloheximide (CHX) was purchased from CST and MG132 and BI6727 were purchased from Selleck.

### Western blot and immunoprecipitation

Cells were washed twice with cold PBS and lysed in RIPA buffer containing proteinase and phosphatase inhibitor cocktails. The protein levels were analyzed by western blotting, and β-actin was used as the loading control. The protein levels were quantified using ImageJ software.

Extracts for immunoprecipitation were prepared with RIPA buffer supplemented with protease inhibitor cocktails. The extracts were incubated with the indicated antibodies on a rotator at 4 °C for 12 h, followed by incubating with protein A/G-magnetic beads (MedChemExpress, Shanghai, China) on a rotator at 4 °C for 1 h. After incubation, beads were washed four times with immunoprecipitation buffer, and boiled in 1× loading buffer. The protein levels were analyzed by western blotting.

### GST pull-down

Recombinant PLK1-His, GST-PLK1, GST-UHRF1 or mutant proteins were expressed in the *E.coli* BL21 strain, and purified. The purified proteins were characterized by Coomassie blue staining. Appropriate amounts of purified GST-tagged protein were mixed with the purified PLK1-His proteins on a rotator at 4 °C for 12 h, and washed four times with the washing buffer. Resin-bound complexes were eluted by boiling and subjected to western blotting.

### In vitro phosphorylation assay

Recombinant human PLK1 or kinase-dead mutant (PLK1-K82R) proteins were expressed in the *E.coli* BL21 strain and purified by IMAC. GST-UHRF1 or its mutant proteins were purified using GST Sefinose Resin. The purified proteins were characterized by Coomassie blue staining. PLK1 kinase active or dead mutant was incubated with UHRF1 or mutant proteins in the kinase buffer with ATP (Cell Signaling Tech, Boston, USA) at 30 °C for 30 min. Reactions were stopped by SDS sample buffer, and samples were then boiled for 5 min and subjected to western blotting.

### In vivo ubiquitination assay

HEK293T cells were transfected with the plasmids for 48 h and then treated with 50 µM MG132 for additional 6 h. The cells were lysed in RIPA buffer containing protease inhibitors and boiled at 100 °C for 10 min. The cell lysates were centrifuged at 12,000 g for 15 min. UHRF1 proteins were immunoprecipitated with anti-His antibody on a rotator at 4 °C for 12 h, and the immune complexes were incubated with protein A/G-magnetic beads on a rotator at 4 °C for 1 h. After being washed, the immunocomplex was subjected to SDS-PAGE and the ubiquitination levels were assessed by immunoblot.

### Chromatin-bound protein isolation

Chromatin-bound proteins were isolated as previously described with minor modifications [44]. Cells were harvested and resuspended in buffer A (10 mM Hepes (pH 7.4), 10 mM KCl, 0.05% NP-40, phosphatase and proteinase inhibitors) and incubated on ice for 20 min. After centrifugation, nuclei were precipitated, washed twice by buffer A, and then lysed in buffer B (10 mM Tris-HCl (pH 7.4), 0.2 mM MgCl, 1% phosphatase and proteinase inhibitors). The mixture was incubated with Triton X-100 (0.1%) on ice for 15 min. After centrifugation, chromatin-protein complexes were precipitated and resuspended in 0.2 N HCl on ice for 20 min. The chromatin-bound proteins were collected in the supernatant by centrifugation and mixed with the same volume of 1 M Tris-HCl (pH 8.0). The solution was boiled and subjected to western blotting.

### DNA dot blot

Genomic DNA was extracted using a genomic DNA purification kit according to the manufacturer’s protocol (CoWin Biosciences, Beijing, China). Genomic DNA was denatured at 95 °C for 10 min, and 100 ng DNA was blotted onto nitrocellulose membrane. After air-drying, DNAs were fixed at 80 °C for 30 min. The membrane was incubated in 5% BSA at room temperature for 1 h, and then with anti-5-methylcytosine antibody at 4 °C overnight. After washing, the membrane was incubated with secondary antibody at room temperature for 1 h and visualized by chemiluminiscence.

### DNA methylation

Global DNA methylation was quantified using the MethylFlashTM Methylated DNA Quantification Kit according to the manufacturer’s protocol (Epigentek Inc, Farmingdale, NY). The kit specifically measured levels of 5-methylcytosine (5-mC) in an ELISA-like microplate-based format. The methylated fraction of DNA was detected using 5mC specific antibodies, and then quantified through an ELISA-like reaction by reading the absorbance in a microplate spectrophotometer at 450 nm (Multiskan-GO, Thermo Fisher Scientific, China). The amount of methylated DNA is proportional to the OD intensity, which can be calculated with the included formulas. The relative methylation status of two different DNA samples or absolute quantification of 5mC was calculated using a standard curve.

### HPLC analysis of 5mC

Genomic DNA was denatured at 95 °C for 10 min, 25 µg of denatured genomic DNA were hydrolyzs 3 h with nuclease P1 (NEB M0660s) at 37 °C and CIP (NEB, #M0520v) was then added and incubated for additional 3 h at 37 °C. Twenty microliters of hydrolyzate were then analyzed using a HPLC system (Shimadzu, SPD-6AV) equipped with a Nacalai cosmosil AR-II C18 column (5 µm, 4.6 × 250 mm). dCMP and 5-me-dCMP were detected by UV-detector at 280 nm wavelength.

### Genome-wide DNA methylation profiling

Genomic DNA was isolated from DU145 cells for bisulfite conversion using the EZ DNA Methylation ^TM^ Kit according to the manufacturer’s protocol (Zymo research, Los Angeles, USA). The DNA was denatured to single-stranded DNA by adding 0.1 N NaOH. After neutralization, the denatured DNA was incubated with whole genome amplification reagent at 37 °C overnight. The DNA fragments were precipitated by adding isopropanol, and precipitated and purified by centrifugation at 4 °C. After air drying, the DNA pellets were re-dissolved in a hybridization buffer reagent. The resuspended DNA was hybridized on the prepared chip in a hybridization oven overnight. The unhybridized and non-specifically hybridized DNA was washed away for subsequent staining and extension. The captured DNA was used as a template, and a single-base extension reaction was performed on the chip. A detectable label group was added to the chip to distinguish DNA methylation. The XC4 reagent was added to the reaction-completed chip, and then dried for 1 h. The processed chips were scanned by a scanner. Because a laser excites the fluorescence of the single-base extension product on the chip, the scanner obtained a high-resolution fluorescence picture. The data were directly imported into the GenomeStudio software for analysis to obtain the methylation data of each sample.

### Senescence-associated β-galactosidase staining

Cellular senescence was assessed by Senescence-Associated β-Galactosidase Staining Kit (Beyotime Biotechnology, Beijing, China) according to the manufacturer’s instructions. The cells were washed twice with PBS, fixed at room temperature for 10–15 min, and incubated with fresh β-gal staining solution at 37 °C overnight. The β-gal-positive cells were monitored under a microscope.

### Statistical analysis

All data were analyzed by Origin 9.0. Results were presented as mean ± SD. Student t-tests was used to analyze the statistical difference of groups. *P < 0.05 and **P < 0.01. *P* < 0.05 was considered as significant.

### Supplementary information

Supplemental material

## Data Availability

Complete microarray datasets for DNA methylation were submitted to the GEO repository under accession number GEO: GSE185400
