# Supplementary material for: PLK1 maintains DNA methylation and cell viability by regulating phosphorylation-dependent UHRF1 protein stability
Source: Cell Death Discov. 2023 Oct 3;9:367. doi: 10.1038/s41420-023-01667-9 (PMC10547799; doi:10.1038/s41420-023-01667-9)
Supplement: Supplementary file 1 — Supplemental material [file 41420_2023_1667_MOESM1_ESM.docx]

**PLK1 Maintains DNA Methylation and Cell Viability by Regulating Phosphorylation-Dependent UHRF1 Protein Stability**

**Yuchong Peng^1,2,3^, Youhong Liu^4,5^, Rirong Zheng^1,2,3^, Yubing Ye^1,2,3^, Yongming Fu^1,2,3^, Linglong Yin^6^, Yingxue Gao^4,5^, Yuxin Fu^4,5^, Xuli Qi^4,5^, Tanggang Deng^1,2,3^, Songwei Zhang^4,5^, Xiong Li^1,2,3,7*^**


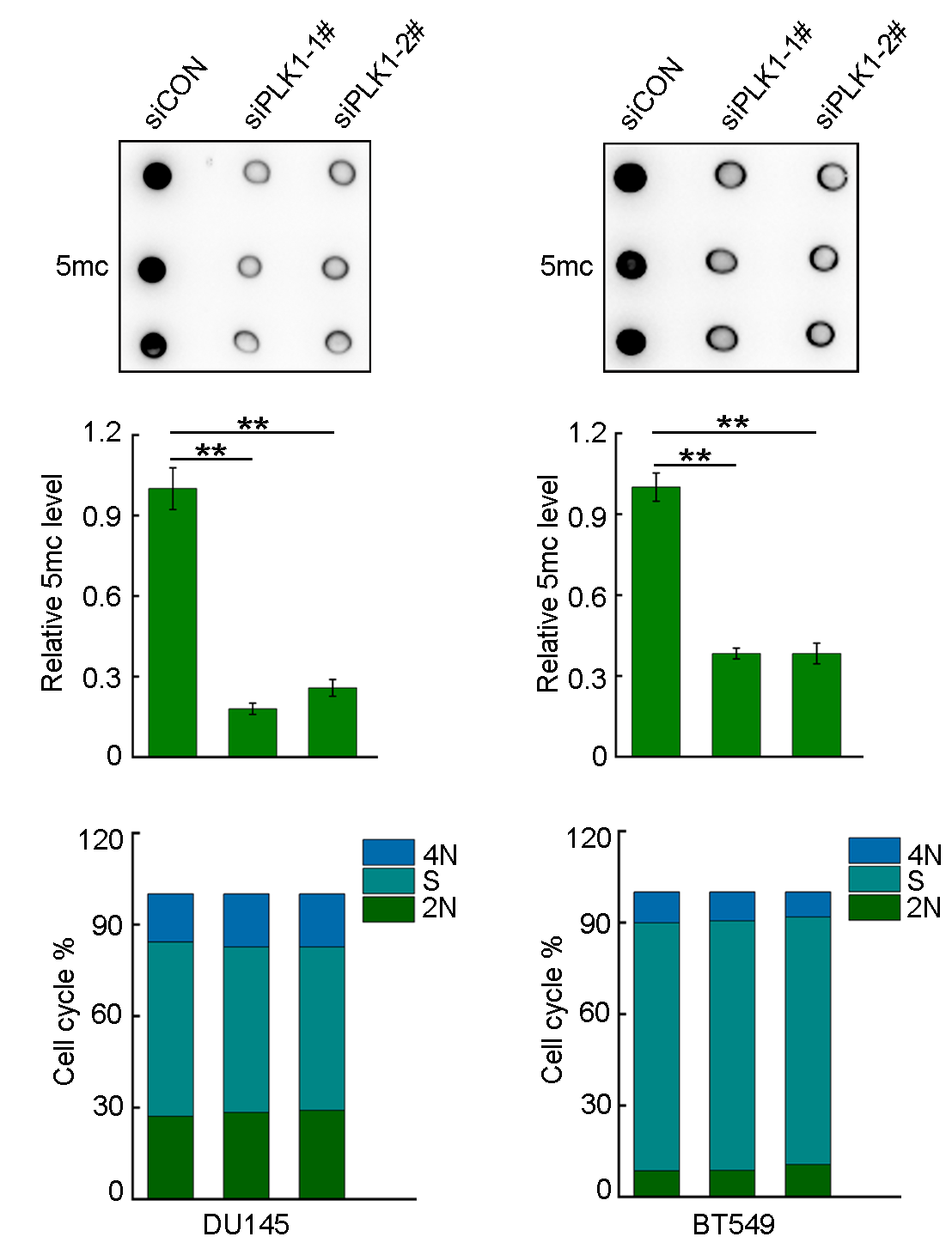


**Fig. S1. PLK1 inhibition decreased the level of DNA methylation.** DU145 and BT549 cells were transfected with either scrambled or two different PLK1 siRNAs, and then cell cycle was synchronized at S phase. The cell cycle phase distribution was analyzed by flow cytometry. The genomic DNA were prepared, and the levels of 5mC were assessed by DNA dot blotting, and the dot intensities were quantified, **P < 0.01.


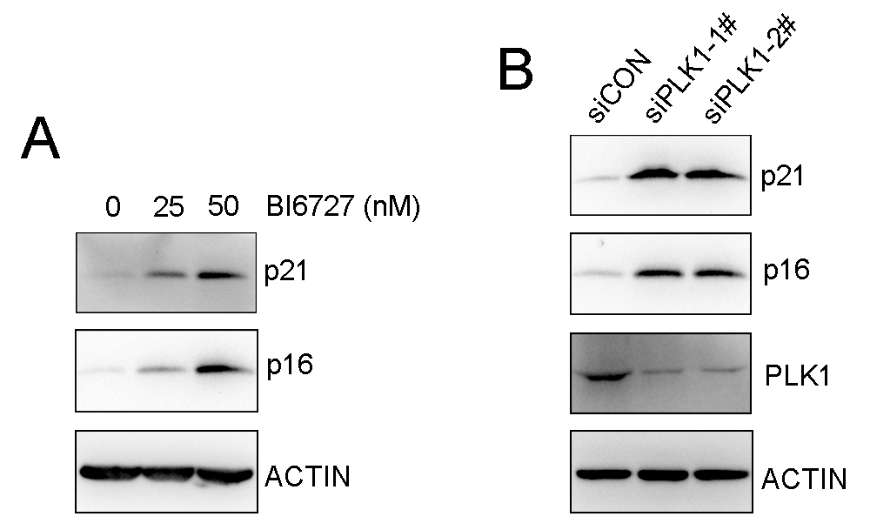


**Fig. S2. PLK1 inhibition elevated the protein levels of p21 and p16.** **A** DU145 cells were treated with different doses of DMSO or BI6727 for 48 h, and the proteins were extracted for immunoblotting. **B** DU145 cells were transfected with either scrambled or two different PLK1 siRNAs, and the proteins were extracted for immunoblotting.


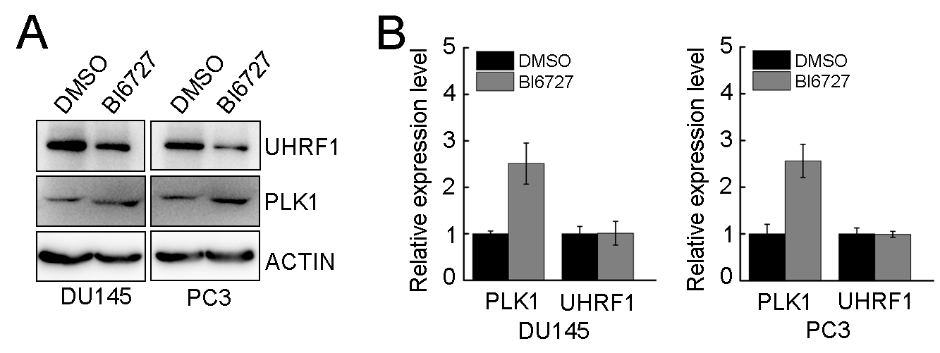


**Fig. S3. PLK1 promotes UHRF1 protein stability.** **A** DU145 and PC3 cells were treated with DMSO or BI6727 (50 nM) for 24 h, and the proteins were extracted for immunoblotting. **B** DU145 and PC3 were treated with DMSO or BI6727 (50 nM) for 24 h. The mRNA levels of *UHRF1* were analyzed by RT-PCR. *ACTIN* was used as an internal control.


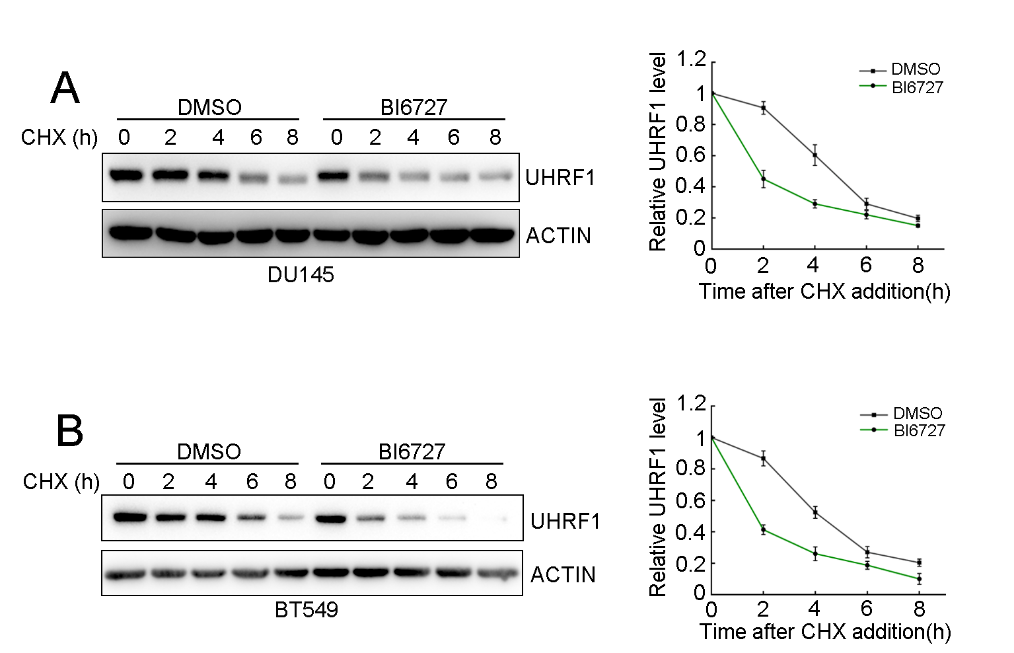


**Fig. S4. PLK1 inhibitor BI6727 promotes UHRF1 protein degradation.** DU145 (**A)** or BT549 (**B)** cells were treated with BI6727 (50 nM) or DMSO for 16 h, and then the protein synthesis was inhibited by cycloheximide (CHX, 50 μg/mL). The proteins were extracted at the indicated time points, and UHRF1 protein levels were analyzed by immunoblotting. ACTIN was used as a loading control. Protein bands were quantified by greyscale analysis. The data was showed as mean±SD from three independent experiments.


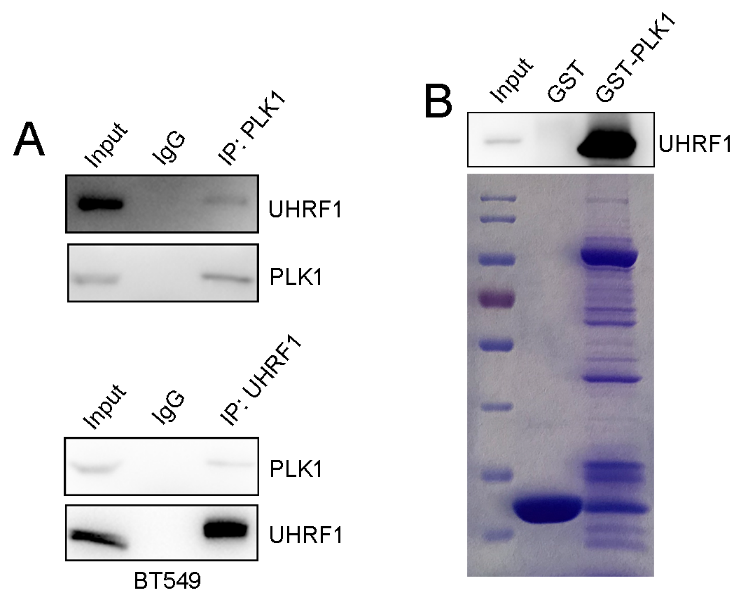


**Fig. S5. UHRF1 protein physically interacts with PLK1.** **A** PLK1 or UHRF1 proteins were immunoprecipitated with anti-PLK1 or anti-UHRF1 antibody in BT549 cell lysates, and the interacting UHRF1 or PLK1 proteins were identified by immunoblotting. **B** The purified recombinant GST or GST-PLK1 proteins were *in vitro* co-incubated with DU145 cell lysates as indicated. The protein interaction between UHRF1 and PLK1 was then assessed by immunoblotting.


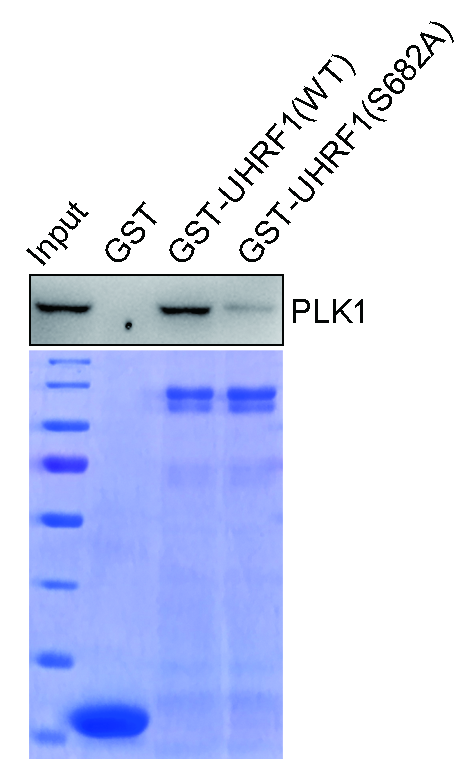


**Fig. S6. The site S682 of UHRF1 is an essential phosphorylation site for the protein physical interaction of UHRF1 and PLK1.** *In vitro* GST pull-down assays were performed by immobilizing GST-UHRF1 wild type or mutants as indicated on glutathione beads. Immobilized GST-UHRF1 proteins were incubated with DU145 cell lysates, and PLK1 protein was assessed by western blotting.


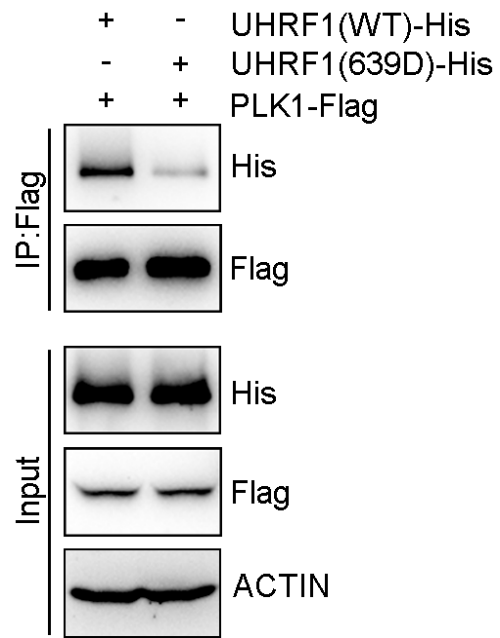


**Fig. S7. CDK1-induced UHRF1 Phosphorylation inhibits the protein interaction of UHRF1 and PLK1.** DU145 cell was transfected with plasmids expressing PLK1-Flag and UHRF1-His wild type or mutants as indicated. PLK1 was immunoprecipitated with anti-Flag antibody, and UHRF1 was assessed with anti-His antibody by western blotting.


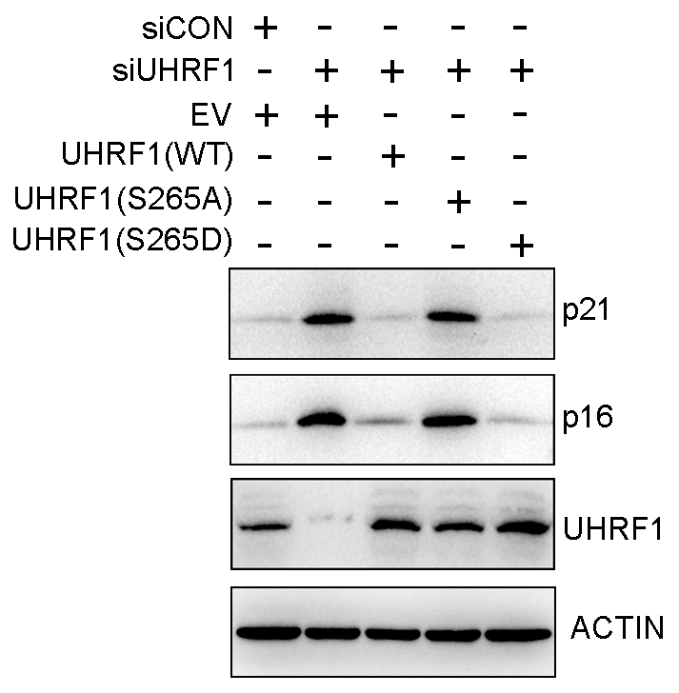


**Fig. S8. Phosphorylation of UHRF1 suppresses p21 and p16 expressions.** DU145 cells were co-transfected with the indicated plasmids and siRNAs for 72 h. The UHRF1, p16 and p21 proteins were assessed by western blotting.

Table S1. Real-time quantitative primer sequences

| Primer name | F primer | R primer |
| --- | --- | --- |
| *PLK1* | CACCAGCACGTCGTAGGATTC | CCGTAGGTAGTATCGGGCCTC |
| *UHRF1* | AGGTGGTCATGCTCAACTACA | CACGTTGGCGTAGAGTTCCC |
| *MAGI2* | AGAATATGACGAACCCGCCC | AAGTTGGGCCTGGGCTTATC |
| *MSX1* | TTGCCACTCGGTGTCAAAGT | AAGGGGACACTTTGGGCTTG |
| *SOX7* | AGCTGTCGGATGGACAATCG | CCACGACTTTCCCAGCATCT |
| *p16* | CGGTCGGAGGCCGATCCAG | GCGCCGTGGAGCAGCAGCAGCT |
| *p21* | ATGGAACTTCGACTTTGTCACC | AGGCACAAGGGTACAAGACAGT |
| *p73* | CAGACAGCACCTACTTCGAC | CTGCTCATCTGGTCCATGG |
